# Prospective Analysis of Confocal Laser Endomicroscopy for Assessment of the Resection Bed for Bladder Tumor

**DOI:** 10.1016/j.euros.2024.11.007

**Published:** 2024-12-05

**Authors:** Ben-Max de Ruiter, Jan E. Freund, C. Dilara Savci-Heijink, Jons W. van Hattum, Marinka J. Remmelink, Theo M. de Reijke, Joyce Baard, Guido M. Kamphuis, D. Martijn de Bruin, Jorg R. Oddens

**Affiliations:** a Department of Urology, Amsterdam UMC, Amsterdam, The Netherlands; b Cancer Center Amsterdam, Amsterdam, The Netherlands; c Department of Biomedical Engineering and Physics, Amsterdam UMC, Amsterdam, The Netherlands; d Department of Pathology, UMC Utrecht, Utrecht, The Netherlands; e Department of Pathology, Amsterdam UMC, Amsterdam, The Netherlands

**Keywords:** Bladder cancer, Diagnostics, Confocal laser endomicroscopy, Cystoscopy

## Abstract

**Background and objective:**

Urothelial bladder cancer (UCB) care requires frequent follow-up cystoscopy and surgery. Confocal laser endomicroscopy (CLE), a probe-based optical technique for real-time microscopic evaluation, has shown promising accuracy for grading of UCB. We investigated the diagnostic accuracy of CLE-based assessment of the surgical radicality of the bladder resection bed (RB).

**Methods:**

We prospectively included 40 participants scheduled for transurethral resection of bladder tumors (TURBT) in two academic hospitals. Exclusion criteria were flat lesions, fluorescein allergy, and pregnancy. We performed CLE of the RB during TURBT. Histopathology of an RB biopsy was the reference test. Results at first cystoscopy 3 mo after TURBT are reported. A panel of two blinded observers evaluated the CLE images. The diagnostic accuracy of CLE for detection of detrusor muscle (DM) and residual tumor (rT) was calculated using 2 × 2 tables.

**Key findings and limitations:**

Histopathology for 22 CLE-matched RB biopsies revealed rT in four cases (18%) and DM in 13 (59%). The quality of CLE imaging was low in four (18%), moderate in 16 (73%), and good in two (9%) cases. CLE was able to correctly predict rT in two of the four cases (50%) identified on histopathology. The sensitivity, specificity, positive predictive value, and negative predictive value were 0.5 (95% confidence interval [CI] 0.07–0.93), 0.83 (95% CI 0.59–0.96), 0.4 (95% CI 0.05–0.85), and 0.88 (95% CI 0.64–0.99) for CLE prediction of rT, and 0.69 (95% CI 0.39–0.91), 0.33 (95% CI 0.07–0.7), 0.6 (95% CI 0.32–0.84), and 0.43 (95% CI 0.1–0.82) for prediction of DM, respectively. Five patients (23%) had rT at 3-mo follow-up; CLE had predicted rT in three, and histopathology had revealed rT in two cases at TURBT.

**Conclusions and clinical implications:**

CLE does not appear to be a reliable tool for detecting rT or DM in the RB after TURBT.

**Patient summary:**

We investigated a special imaging technique called confocal laser endomicroscopy (CLE) for checking the bladder after surgery for bladder cancer in a group of 40 patients. CLE results were compared to traditional biopsy results and the patients were checked after 3 months. CLE was not very reliable in detecting any remaining cancer (only 50% accurate) or important muscle tissue in the surgical area, and the quality of the images varied. While CLE shows some promise, it is not currently a dependable method for evaluating the bladder after bladder cancer surgery.

## Take Home Message

According to our study results, confocal laser endomicroscopy does not appear to be a reliable tool for detecting residual tumor or detrusor muscle in the resection bed after transurethral resection of bladder tumor and cannot replace repeat biopsy.

## 1 Introduction

Bladder cancer (BC) is ranked among the top ten most common forms of cancer globally and has the highest lifetime treatment cost per patient of all cancers [1], [2]. Current diagnostic and treatment paradigms encompassing repeat cystoscopies and surgical resections are both time-intensive and costly [3]. Emerging alternative approaches, such as confocal laser endomicroscopy (CLE), to streamline the diagnostic process and potentially reduce associated costs are currently under investigation.

Diagnosis and staging of BC typically occur during cystoscopy and are confirmed via transurethral resection of bladder tumor (TURBT). The excised specimen is subjected to histopathological evaluation for definitive grading and staging. More than 90% of BC cases involve urothelial carcinoma of the bladder (UCB) [4]. A critical demarcation in treatment strategies is established based on whether the cancer has invaded the muscle layer of the bladder (MIBC) or is confined to the bladder mucosa, known as non–muscle-invasive bladder cancer (NMIBC). MIBC has a higher risk of progression and metastasis in comparison to NMIBC. NMIBC is stratified into categories regarding the risk of progression or metastasis [5].

Several systematic reviews have suggested that for high-risk tumors, a second TURBT often reveals a significant likelihood of residual tumor and potential muscle invasion [6], [7]. A second TURBT is therefore associated with better recurrence-free survival [8]. Consequently, BC guidelines recommend a second TURBT within 2–6 wk following the initial resection in cases with T1 disease [5], [9]. This escalates both the clinical costs and the burden on the patient. By contrast, a Danish national cohort study found that 35.9–52.9% of TURBT samples were devoid of neoplasia on histopathological evaluation, so an excessive number of surgeries were unnecessary [10].

Confocal laser endomicroscopy (CLE) is a probe-based optical imaging technique that appears to be promising for real-time histopathological assessment [11]. Sonn et al [12] were the first to report successful in vivo application of CLE during TURBT. Using intravenous fluorescein, the authors were able to identify muscularis propria and fat lobules in the resection bed (RB). Their results for RB assessment were reproduced by others and it has also been suggested that CLE improves the therapeutic efficacy of TURBT [13], [14].

There has been no evaluation of the diagnostic accuracy of CLE for RB assessment. We therefore conducted the CLETUR trial, which had two objectives. For the first objective, we have reported that low-definition probe-based CLE during flexible cystoscopy appears to be unable to grade UCB [15]. Here we report on the second objective, which was to evaluate the diagnostic accuracy of high-definition CLE for RB assessment following TURBT.

## 2 Patients and methods

### 2.1 Study design and patients

CLETUR is a prospective, multicenter pilot trial with a paired study design with two primary objectives (NCT05273593). Here we report on the diagnostic accuracy of CLE assessment of the surgical radicality of the RB in comparison to histopathology of a biopsy specimen collected from the RB during TURBT [15]. A Cystoflex ultra-high definition UHD-R probe (Mauna Kea Technologies, Paris, France; imaging depth 50–65 μm, field of view 240 μm, lateral resolution 1 mm) was used for RB assessment. The study results are reported in accordance with the Standards for Reporting of Diagnostic Accuracy Studies guidelines [16]. Eligibility criteria for patient inclusion consisted of a planned TURBT procedure for cystoscopically identified exophytic bladder lesions. Patients with flat lesions (eg, carcinoma in situ) were excluded from the study. Additional exclusion criteria were a known allergy to fluorescein and pregnancy. Written informed consent was obtained from all participants. Ethics approval for the study protocol was granted by the institutional review board (IRB) of each participating center, in accordance with the guidelines for good clinical practice (IRB reference 2019_197).

### 2.2 Study procedures

Subsequent to induction of anesthesia but before TURBT, a Foley catheter was inserted into the bladder, which was then instilled with a solution of 200–400 ml of 0.1% fluorescein that was left in the bladder for 5 min to allow staining of the extracellular matrix of the bladder mucosa. After catheter removal, CLE imaging of the tumor of interest (TOI) was conducted using a low-power 488-nm laser system (Cellvizio 100 series; Mauna Kea Technologies). After CLE imaging, the TOI was excised in a piecemeal fashion using a 27Fr resectoscope with 0° optics in accordance with standard clinical protocols. When TURBT was complete, 2.5 ml of 10% fluorescein was administered intravenously, and three 20-s recordings from three distinct regions on the RB were acquired at a frame rate of 8–12 frames/s, thereby capturing the RB cellular microarchitecture using the working channel of the resectoscope. Then RB biopsy was conducted as the gold standard for comparison with CLE imaging. If no histopathological biopsy could be obtained, the patient was excluded from the final analysis. The result of the first cystoscopy after 3 mo in NMIBC cases was also recorded for assessment of potential residual tumor in the RB that could have been missed during CLE. Histopathological analyses were carried out in compliance with standard clinical protocols by an experienced uropathologist (C.D.S.-H.) who remained blinded to the CLE images.

### 2.3 CLE image evaluation

After a post-acquisition “washout” period of 12 wk, the CLE images were assessed in an offline setting by two experienced CLE observers (C.D.S.-H. and J.E.F.) who were blinded to the clinical information [17], [18]. The raw image quality of CLE images of the RB was rated for each case using a 3-point Likert scale (poor, moderate, or high), followed by a descriptive analysis of the type of tissue, including the extent of cautery artifacts. Finally, the diagnostic accuracy of CLE for detection of connective tissue, detrusor muscle, and tumor was calculated. The CLE images were assessed according to previously validated features for differentiating between benign and malignant lesions [17]. If both observers rated the CLE images as “insufficient data”, the CLE images were classified as nondiagnostic. These results are reported, but the cases were excluded from the analysis. Histopathology was used as the reference test for calculation of the CLE diagnostic accuracy. Outcomes of the first cystoscopy at 3-mo follow-up are also reported as a surrogate measure for complete resection [19]. Cellvizio Viewer software (Mauna Kea Technologies) was used for frame-by-frame analysis.

### 2.4 Statistical analysis

The sample size for this pilot study was initially arbitrarily projected to be 60 measurements, which was based on the availability of CLE probes. However, owing to restrictions imposed during the COVID-19 pandemic, the IRB was consulted and the total amount of inclusions was reduced to 40 measurements. Descriptive statistics are reported for baseline variables. The diagnostic accuracy, sensitivity, specificity, negative predictive value, and positive predictive value for CLE were calculated using 2 × 2 tables. All analyses were conducted in SPSS version 28 (SPSS Inc., Chicago, IL, USA).

## 3 Results

### 3.1 Patient characteristics

**Fig. 1:**
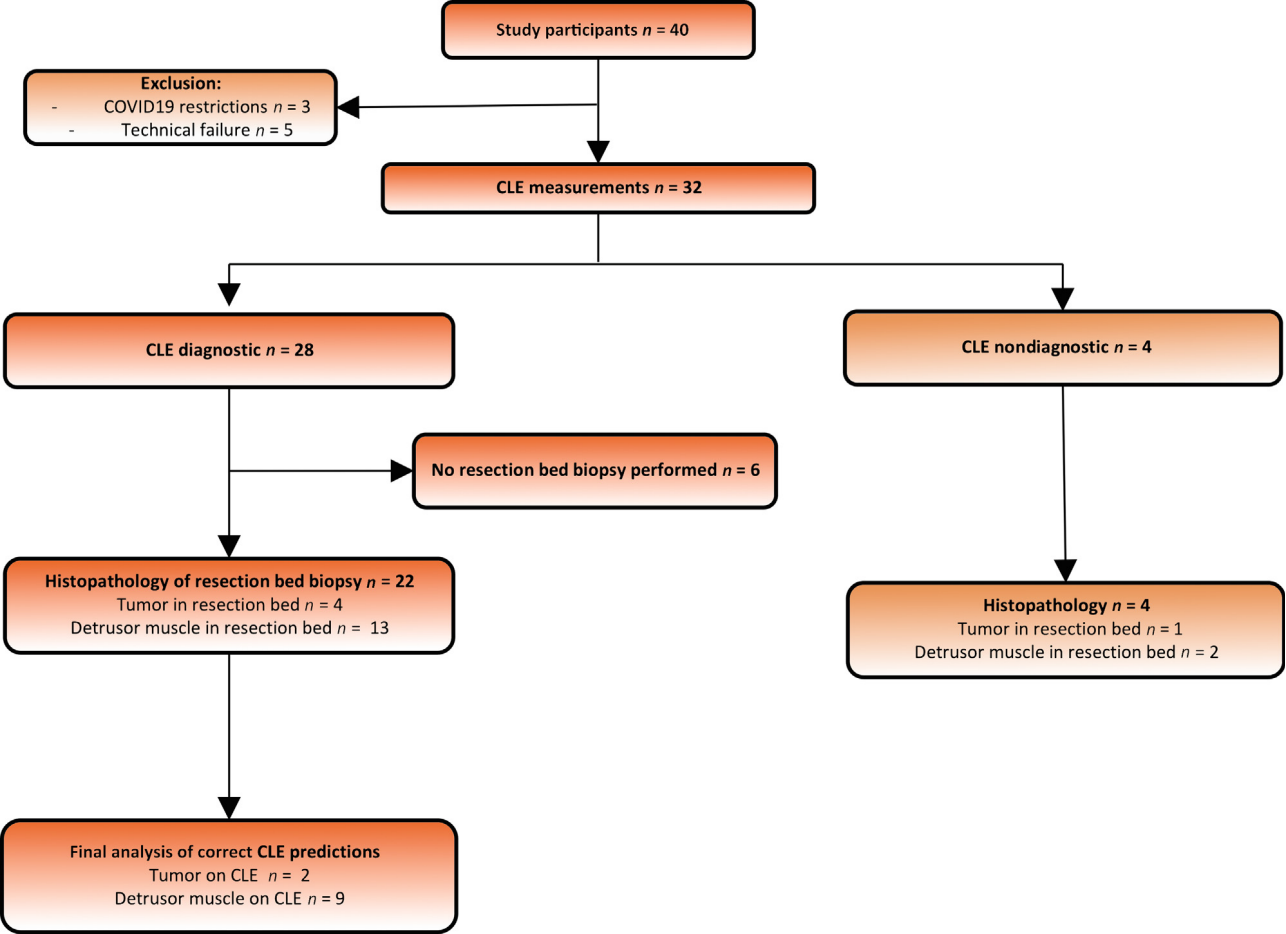
Study flowchart. CLE = confocal laser endomicroscopy.

Between January 2020 and November 2021, 40 participants were enrolled in two academic hospitals in The Netherlands. Figure 1 shows the study flowchart. Three patients were excluded for logistical reasons related to restrictions on research during the COVID-19 pandemic. In five patients the tumor RB could not be reached owing to technical failures: the probe could not reach the RB in three cases, and there was angulation near the bladder trigone in two cases. In six patients, RB biopsy could not safely be obtained owing to the depth of the primary resection. Baseline characteristics are listed in Table 1. Benign lesions included reactive atypia (*n* = 1), nephrogenic adenoma (*n* = 1), and inverted papilloma (*n* = 1).

**Table 1:** Patient and tumor characteristics for the study cohort (*n* = 22)

Parameter	Result
Median age, yr (IQR)	72 (62–76)
Male, n (%)	16 (73)
Tumor location, *n* (%) a	
Bladder trigone	6 (27)
Anterior wall	1 (5)
Posterior wall	7 (32)
Lateral	16 (73)
Bladder dome	4 (18)
Tumor size <3 cm, *n* (%)	19 (86)
Tumor stage, *n* (%)	
pTa	13 (59)
pT1	3 (14)
pT2	3 (14)
Benign	3 (14)
Histopathology, *n* (%)	
Pure UCB	19 (86)
Benign	3 (14)
High grade, *n* (%) b	11 (58)

IQR = interquartile range; UCB = urothelial carcinoma of the bladder.

a Multiple locations were possible.

b World Health Organization 2004 classification.

### 3.2 Imaging quality

**Fig. 2:**
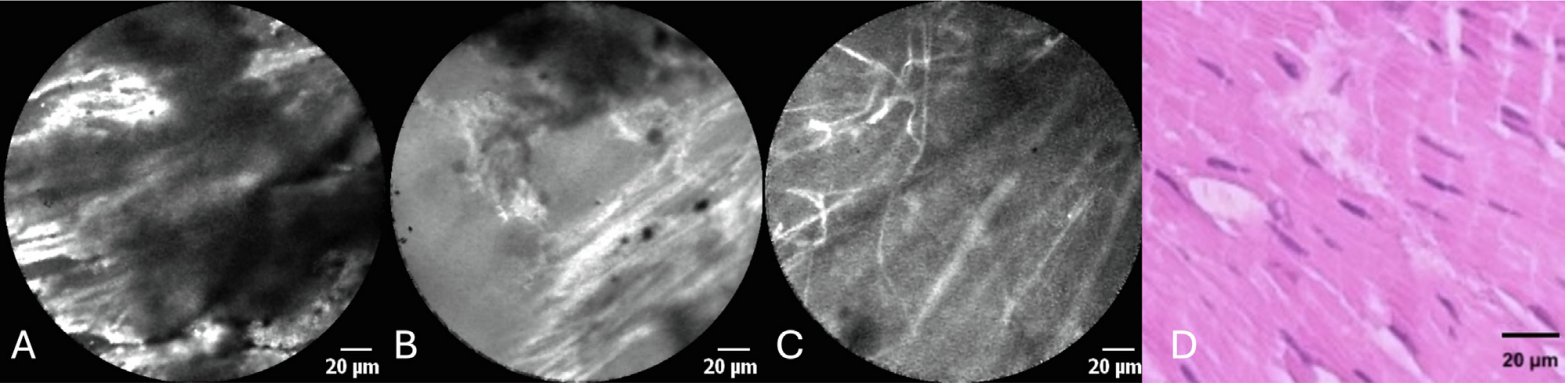
CLE images and H&E-stained section of a resection bed specimen. (A) Low-quality CLE image with prominent cautery artifacts. (B) Moderate-quality CLE image showing muscle fibers and fewer cautery artifacts. (C) High-quality CLE image showing muscle fibers, with no cautery artifacts evident. (D) H&E-stained section of a biopsy specimen from the resection bed showing smooth muscle fibers. CLE = confocal laser endomicroscopy; H&E = hematoxylin and eosin.

According to the panel, the quality of CLE images of the RB was low in four (18%), moderate in 16 (72%), and good in two (9%) cases. Figure 2 shows CLE images of differing quality. Supplementary Video 1 shows CLE images of a grade 3 pT1 tumor and Supplementary Video 2 shows the corresponding RB after radical resection. Cautery artifacts (Fig. 2A,B) were present in ten cases (46%) and corresponded to lower image quality.

### 3.3 Diagnostic accuracy

Table 2, Table 3 show 2 × 2 tables comparing CLE prediction of the presence of UCB and detrusor muscle versus the RB biopsy results. Table 4 lists diagnostic performance results for CLE. CLE was able to correctly predict residual tumor in two of the four cases (50%; one high-grade pT1 and one pT2) identified via RB biopsy. In three cases (one low-grade pTa, one high-grade pT1, and one high-grade pTa) the CLE expert panel observed tumor, but no tumor was identified on RB biopsy. Among these three cases, evaluation at 3-mo cystoscopy showed residual tumor in one patient (high-grade pTa; TURBT prompted by the 3-mo cystoscopy confirmed that the residual tumor was high-grade pTa), corresponding to incomplete TURBT during the CLE procedure. Five out of 22 cases showed residual UCB at 3-mo follow-up. In three of these cases, CLE predicted UCB in the RB. In two of these cases, the RB biopsies were positive.

**Table 2:** Cross-comparison (2 × 2 table) of histopathology results versus CLE prediction of UCB in the resection bed

Histopathology	CLE prediction		Total, *n* (%)
result	UCB	No UCB	
UCB	2	2	4 (18)
No UCB	3	15	18 (82)
Total, *n* (%)	5 (23)	17 (77)	22 (100)

CLE = confocal laser endomicroscopy; UCB = urothelial carcinoma of the bladder.

**Table 3:** Cross-comparison (2 × 2 table) of histopathology results versus CLE prediction of DM in the resection bed

Histopathology	CLE prediction		Total, *n* (%)
result	DM	No DM	
DM	9	4	13 (59)
No DM	6	3	9 (41)
Total, *n* (%)	15 (68)	7 (32)	22 (100)

CLE = confocal laser endomicroscopy; DM = detrusor muscle.

**Table 4:** Diagnostic performance of CLE

Tissue type	Cases, *n* (%)		Performance metric (95% CI)			
in RB-Bx	Actual	CLE prediction	Sensitivity	Specificity	PPV	NPV
UC	4 (18)	5 (23)	0.5 (0.07–0.93)	0.83 (0.59–0.96)	0.4 (0.05–0.85)	0.88 (0.64–0.99)
Connective tissue	8 (36)	8 (36)	0.25 (0.03–0.65)	0.57 (0.29–0.82)	0.25 (0.03–0.65)	0.57 (0.29–0.82)
Detrusor muscle	13 (59)	15 (68)	0.69 (0.39–0.91)	0.33 (0.07–0.7)	0.6 (0.32–0.84)	0.43 (0.1–0.82)

CI = confidence interval; CLE = confocal laser endomicroscopy; NPV = negative predictive value; PPV = positive predictive value; RB-Bx = resection bed biopsy; UC = urothelial carcinoma.

## 4 Discussion

Residual disease and understaging are prevalent after initial TURBT. Absence of detrusor muscle in the specimen is associated with significantly higher risk of residual disease and tumor understaging [6]. A second resection is therefore advised in the American Urological Association and European Association of Urology guidelines in cases of incomplete resection and the absence of detrusor muscle, with the exception of low-grade/grade 1 Ta disease [5], [9]. Despite the introduction of new visualization techniques for TURBT, none has led to omission of this second resection.

We evaluated the diagnostic accuracy of in vivo high-definition CLE to assess tissue types in the RB during TURBT. We were able to match CLE with histological biopsy results in 22 out of 40 cases. Nondiagnostic procedures and technical failures of the CLE system were prevalent and led to a dropout rate of 23%. We also found low concordance of CLE with RB histopathology. Cautery artifacts after diathermic resection were prevalent and predominantly contributed to lower CLE image quality.

The sensitivity and specificity for residual UCB were lower than in studies evaluating CLE for primary diagnosis of UCB [20], [14]. Cautery artifacts and the flat aspect of the RB appear to be the main contributors to the lower diagnostic performance. Previous research showed that differentiation of flat lesions appears to be difficult with CLE, potentially because of the imaging depth and angulation required to reach lesions [17], which impedes consistent measurement of the required field of view and imaging depth.

In addition, we found one CLE-positive case for which the RB biopsy was negative for remaining tumor, but the first cystoscopy at 3 mo did reveal residual tumor. Moreover, only two of the four patients with residual UCB in the RB biopsy had residual tumor on follow-up cystoscopy. These findings highlight the potential sampling bias of RB biopsy.

Detrusor muscle in the RB biopsy was present in 13 cases (59%). Although detrusor muscle was seen on CLE in 15 cases (68%), the specificity of CLE for detrusor muscle was surprisingly low (0.33, 95% confidence interval 0.07–0.7). Although this may suggest sampling bias, another explanation could be the force needed for the CLE probe to obtain an adequate fluorescent signal or cautery artifacts. Furthermore, warm biopsies using bipolar TURBT were obtained, which may have led to a reduced biopsy thickness and further thermal damage to the specimen, affecting histopathological identification of smooth muscle [21].

As mentioned, cautery artifacts leading to reduced uptake of intravenous fluorescein and blocking of the confocal laser were prevalent and a major reason for lower image quality. Fluorescein is a nonspecific dye that stains the extracellular matrix. It is a contrast agent with US Food and Drug Administration approval for intravenous and topical administration. We used intravenous application, as previous work suggested that fluorescein can diffuse through the transitional epithelium into the lamina propria and thus might provide better RB imaging [12].

To the best of our knowledge, no other fluorophore is currently under investigation in combination with CLE. Marien et al [22] tested hexaminolevulinate (HAL) but found that HAL-CLE–only histological analysis was not possible because HAL is mostly cytoplasmic and is poor at revealing details of the cellular architecture. In an ex vivo study, Pan et al [23] used CLE with the molecular marker CD-47 as a fluorescent agent. However, no in vivo results have been reported to date.

Other imaging techniques such as optical coherence tomography (OCT) may be more suitable for evaluation of flat lesions. OCT has shown high sensitivity for assessment of muscle invasion and lamina propria invasion. Conversely, it has low specificity for BC and its diagnostic accuracy might further be hampered by cautery artifacts [24], [25]. Another emerging technique is multiparametric cystoscopy, which combines different imaging modalities [26]. Although the technique is feasible and promising, the results have not been reproduced in larger samples and require further validation.

Strengths of our study are the direct and prospective histopathological comparison with CLE imaging, the use of two experienced CLE observers, and the evaluation at 3-mo cystoscopy. Limitations include the high exclusion rate and the relatively low prevalence of residual UCB in RB biopsies.

According to the study findings, CLE of the RB is unable to assess remaining tissue during primary TURBT and can be omitted. Future research into CLE for UCB should focus on primary diagnosis and follow-up of exophytic lesions, combining CLE with other fluorophores or molecular markers or other imaging techniques, possibly in a multispectral manner.

## 5 Conclusions

In this small study, the diagnostic performance of high-definition CLE for RB assessment after TURBT was suboptimal. Further research is necessary to improve the diagnostic yield and performance of CLE in this setting.

  ***Author contributions***: Ben-Max de Ruiter had full access to all the data in the study and takes responsibility for the integrity of the data and the accuracy of the data analysis.

  *Study concept and design*: de Ruiter, Freund, de Bruin, de Reijke, Oddens.

*Acquisition of data*: de Ruiter, van Hattum, Kamphuis, Savci-Heijink, Baard.

*Analysis and interpretation of data*: de Ruiter, van Hattum, Freund.

*Drafting of the manuscript*: de Ruiter, Freund, Oddens, Remmelink.

*Critical revision of the manuscript for important intellectual content*: de Bruin, de Reijke, Savci-Heijink, Baard, Kamphuis.

*Statistical analysis*: de Ruiter, van Hattum, Freund.

*Obtaining funding*: Oddens, de Bruin, de Reijke.

*Administrative, technical, or material support*: de Bruin, de Reijke.

*Supervision*: de Reijke, de Bruin, Freund.

*Other*: None.

  ***Financial disclosures:*** Ben-Max de Ruiter certifies that all conflicts of interest, including specific financial interests and relationships and affiliations relevant to the subject matter or materials discussed in the manuscript (eg, employment/affiliation, grants or funding, consultancies, honoraria, stock ownership or options, expert testimony, royalties, or patents filed, received, or pending), are the following: None.

  ***Funding/Support and role of the sponsor*:** Ben-Max de Ruiter has received a research grant from the Dutch Cancer Society. Ben-Max de Ruiter and Jons W. van Hattum have received a research grant from the “Cure for Cancer” Foundation. Mauna Kea Technologies supplied the CLE probes used in this study. The sponsors did not play any direct role in the study.
